# Mechanisms of follicular atresia: focus on apoptosis, autophagy, and ferroptosis

**DOI:** 10.3389/fendo.2025.1603467

**Published:** 2025-09-23

**Authors:** Tongqing Zhang, Menghan Lin, Chuangui Wang, Jiaqi Zhou

**Affiliations:** School of Life Sciences and Medicine, Shandong University of Technology, Zibo, China

**Keywords:** follicle atresia, apoptosis, autophagy, ferroptosis, premature ovarian failure

## Abstract

Follicular atresia is a critical physiological process that ensures the selection of high-quality oocytes by eliminating non-viable follicles. In women, 99.9% of follicles undergo atresia naturally, the premature or dysregulated atresia follicle can lead to pathological conditions such as polycystic ovary syndrome (PCOS) and premature ovarian failure (POF). Recent studies highlight the roles of apoptosis, autophagy, and ferroptosis in regulating follicular atresia. This review integrates molecular mechanisms of programmed cell death with follicular dynamics, emphasizing how aberrant atresia disrupts reproductive health. We discuss therapeutic strategies targeting these pathways to mitigate pathological atresia, offering insights into preserving ovarian reserve and improving fertility outcomes.

## 1 Introduction

In mammals, 99.9% of follicles die by atresia, an irreversible physiological feature that is important for maintaining ovarian homeostasis and selecting viable oocytes (1). A massive reduction in the number of oocytes occurs during the germline cyst breakdown and formation of primordial follicles, with only 20% of the follicles surviving by the time of birth, and many molecules are involved (2). Follicular atresia persists in the ovaries after birth, and in humans, only about 400 follicles develop, mature, and ovulate normally during the reproductive years (3). Follicular atresia is a process of degenerative death of oocytes and somatic cells and can occur at all stages of follicular development. Among them, primordial follicular atresia is rare, and primary follicular atresia is the most common (4). The morphological features are: nuclear condensation of the oocyte, chromosomal and cytoplasmic lysis, reduction of the granulosa cell layer, hypertrophy of the follicular membrane cells, and the appearance of lipids in the cytoplasm, luteinization, and scattering in connective tissues, which constitute the so-called “interstitial glands (5, 6).”Subsequently, the oocytes degenerate and the granulosa cells and follicular membrane cells evolve into fibrous bodies that can be absorbed by the follicular mesenchyme (7). At the same time, granulosa cells in atretic follicles produce less estrogen, increased progesterone production, decreased number of gonadotropin receptors, and enhanced expression of IGF-binding proteins (8–10). In addition, decreased expression of the gap junction protein connexin43 and enhanced expression of the sulfated glycoprotein sP-2 are observed in atretic follicles (11, 12). Therefore, exploring the molecular mechanisms regulating follicular atresia not only helps to elucidate the cause of massive follicle loss under physiological conditions, but also is of great significance in revealing the onset mechanism of premature ovarian aging and slowing down ovarian aging.

Programmed cell death is generally defined as a spontaneous form of cell death regulated by a variety of biomolecules (11, 13). Apoptosis was widely recognized by the general public as the main mode of programmed cell death, and more modes of programmed cell death have gradually emerged with the intensive study of cell biology, such as autophagy and ferroptosis (8, 14, 15). Programmed cell death plays an important role in maintaining homeostasis in the body, sustaining normal physiological functions, host defense against pathogens, cancer, and a wide range of other pathological processes (16). During follicular formation and development, follicular atresia is closely associated with programmed cell death within the follicle. There is a close interaction between the oocyte and other somatic cells within the follicle that collectively leads to the development of follicular atresia (17).

Physiological follicular atresia eliminates defective follicles, ensuring optimal reproductive capacity. Follicular atresia occurs at all developmental stages, from primordial to antral follicles, driven by complex interactions between oocytes and somatic cells (3). Morphologically, atresia involves oocyte degeneration, granulosa cell apoptosis, and follicular remodeling (4). Dysregulation of phosphatidylinositol-3-kinase/AKT serine/threonine kinase (PI3K/AKT) and mammalian target of rapamycin (mTOR) signaling pathways and oxidative stress exacerbate pathological atresia, linking it to PCOS and chemotherapy-induced ovarian damage (5, 6). We can use this as an entry point to inhibit follicular atresia by regulating the programmed death of follicular cells using drugs or other means. The goal is to prevent premature ovarian failure, maintain the number and quality of oocytes in the follicles, and prolong reproductive age as well as improve fertility.

## 2 Follicular atresia across primordial and developmental follicles stages

**Figure 1:**
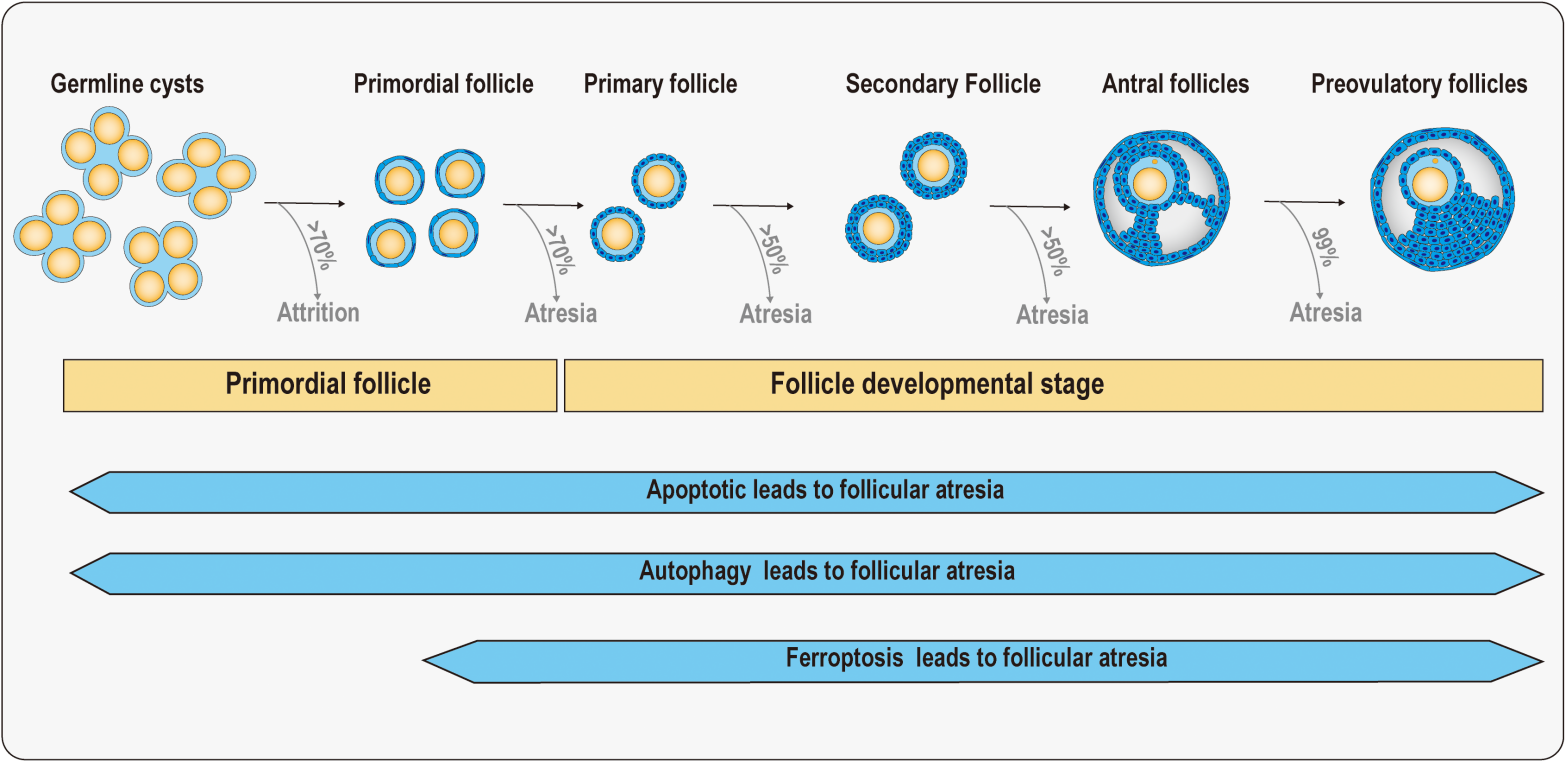
Schematic diagram of follicular atresia at primordial follicle and follicle developmental stage. In the ovaries of a human fetus at 20 weeks of age, there are approximately 7 million germ cells. There are approximately 1–2 million primordial follicles in the ovaries of newborns in the early stages, but by the age of 7, only about three hundred thousand primordial follicles remain. More than 50% of follicles will experience atresia in both primary and secondary follicular stages. Before ovulation, 99% of antral follicles will undergo atresia, with only one or a few follicles ovulating. When the follicle reserve is depleted, the ovaries rapidly age as women enter menopause. Meanwhile, apoptosis, autophagy, and ferroptosis have been found to be associated with follicular atresia.

Follicular atresia is a phenomenon in which a follicle stops growing and begins to degenerate during follicular development for a variety of reasons. This process is a natural part of the ovarian cycle, but when follicular atresia occurs in excess or at inappropriate times, it can lead to fertility problems (14). Follicular atresia can be achieved by inhibiting the development of most follicles, ensuring the quality of oocytes as well as the production of superior offspring. After the formation of primordial follicles, follicular atresia is accompanied by various stages of follicular development, including apoptosis, autophagy, and ferroptosis, all of which are involved in regulating follicular atresia (**Figure 1**).

### 2.1 Primordial follicle

At this stage, the atresia of the primordial follicle is mainly related to the signal imbalance between oocytes and granulosa cells. Some studies have confirmed this. For example, glycogen synthase kinase-3 beta (GSK3β) plays a crucial role in the survival of oocytes during prophase I of meiosis. By regulating the transcriptional activity of β-catenin, GSK3β influences the spatiotemporal expression pattern of P63, ensuring the normal progression of meiotic prophase. Furthermore, in mice with germ cell-specific knockout of *Gsk-3β*, a significant increase in oocyte apoptosis was observed, accompanied by a marked reduction in the number of primordial follicles (18). Specificity protein 1 (SP1) regulates the formation and activation of primordial follicles. Deletion of *Sp1* resulted in impaired breakdown of germ cell cysts and reduced primordial follicle pool (19). The mechanistic target of rapamycin complex 1/KIT ligand (mTOR1/KITL) pathway is also involved in this process. mTORC1 activates KITL in granulosa cells, promoting survival of the primordial follicle and inhibiting its premature activation (20). Studies have also revealed that iroquois homeobox 3 (IRX3) and iroquois homeobox 5 (IRX5) are key factors for the crosstalk between oocytes and granulosa cells (21). A recent study also found that protein phosphatase 4 regulates the autophagy of oocytes to maintain the survival of primordial follicles (22). In conclusion, the interactions between oocytes and granulosa cells are crucial for follicle survival and atresia.

Recent investigations have further identified neurotrophins (NTs) as potential regulators of primordial follicle activation (23). Inhibition of nerve growth factor (NGF) or its receptor neurotrophic receptor tyrosine kinase 1 (NTRK1) significantly reduces oocyte numbers and impairs primordial follicle numbers in mouse ovaries. Conversely, supplementation with connective tissue growth factor (CTGF) markedly increases primordial follicle numbers *in vitro* (24). These findings collectively highlight the importance of coordinated and efficient communication between oocytes and granulosa cells during folliculogenesis. Such cellular interactions are essential for optimizing follicular development, maintaining the balance between primordial follicle survival and atresia, and ensuring the provision of high-quality oocytes for reproductive success.

Furthermore, *in vitro* culture of embryonic mouse ovaries with transforming growth factor beta 1 (TGF-β1) suppresses primordial follicle activation and reduces the primordial follicle pool, whereas supplementation with SD208, a TGF-β1 inhibitor, significantly promotes primordial follicle development (25). These findings collectively highlight the critical roles of signaling molecules and their regulatory networks in primordial follicle assembly and maintenance.

As the most critical component of primordial follicles, oocytes secrete some specific molecules that are necessary for the activation or atresia of primordial follicles, including factor in the germline alpha (FIGα), newborn ovary homeobox gene (NOBOX), spermatogenesis and oogenesis specific basic helix-loop-helix 1 and 2 (SOHLH1 and 2), and lim homeobox 8 (LHX8) (26–29). Research has demonstrated that *Figα* knockout mice fail to form primordial follicles, leading to a rapid loss of oocytes postnatally, while male development remains unaffected (30). Consequently, the ovarian failure phenotype in these mice positions FIGα as a candidate gene for human premature ovarian failure. Additionally, homozygous deletion of *Nobox* results in follicle atresia after birth (31).

### 2.2 Follicle developmental stage

The development and ovulation of oocytes within follicles in mammals are tightly regulated by gonadotropins. Follicle-stimulating hormone (FSH) plays an indispensable role in the selection of follicles and the development of dominant follicles. Insufficient FSH secretion or reduced sensitivity of follicles to FSH can lead to follicular atresia. The differentiation of follicular sensitivity is likely the result of a combination of endocrine, paracrine, and autocrine factors; however, the precise molecular mechanisms underlying this process remain largely unclear. A recent study revealed that lysine-specific demethylase 1 (LSD1) can regulate granulosa cell autophagy levels, thereby participating in FSH-mediated antral follicle formation and fate determination (32). Nevertheless, the critical scientific question of how LSD1 regulates follicle stimulating hormone receptor (FSHR) to enhance follicular sensitivity to antrum formation remains unanswered. Although lots of follicles ultimately undergo atresia, under physiological conditions, the arrival of FSH can promote follicular development and survival. Research has demonstrated that, in the presence of energy stimulation, the FSHR-mTOR-HIF1 signaling pathway can rescue follicles from atresia (33). However, the origin of the energy differences driving follicular atresia and how a cohort of follicles can transmit FSH signals into their microenvironment remain unresolved. These important questions warrant further investigation.

During follicular development, the abnormal development of granulosa cells is often the main cause of follicular atresia. The study found that forkhead box L2 (FOXL2) can negatively regulate splicing factor 1 (SF1) to activate cytochrome P450 family 17 subfamily A member 1 (CYP17A1) transcription, leading to granulosa cell development defects, and consequently inducing follicular confinement (34). The imbalance of BCL2 apoptosis regulator/BCL2 associated X (BCL-2/BAX) in granulosa cells and the overexpression of BAX lead to the release of cytochrome C by mitochondria, activation of Caspase-3 and apoptosis (17). The PI3K/AKT pathway is also involved in regulation. FSH and IGF-1 activate PI3K/AKT, phosphorylate forkhead box O3, (FOXO3) and inhibit its nuclear translocation, reducing the expression of pro-apoptotic genes such as fas ligand (FASLG) (35). In addition, growth differentiation factor 9 (GDF9) inhibits granulosa cell apoptosis through SMAD family member 3 (SMAD3) and collaborates with PI3K/AKT to enhance survival signals (36). At secondary and antral follicles stage, besides apoptosis, autophagy and ferroptosis pathways are gradually involved in follicular atresia. Autophagy plays a dual role. Protective autophagy can remove damaged organelles and maintain granulosa cell homeostasis (37). Excessive autophagy, such as nutrient deficiency or BPA (Bisphenol-A) exposure, activates adenosine 5’-monophosphate (AMP)-activated protein kinase/mammalian target of rapamycin/Unc-51 like autophagy activating kinase 1 (AMPK/mTOR/ULK1) pathways, upregulates microtubule associated protein 1 light chain 3 beta (LC3B), beclin 1 (BECN1), and accelerates follicular atresia (38). Ferroptosis generally occurs under specific circumstances, such as when chemotherapy drugs (such as cyclophosphamide) induce lipid peroxidation through heme oxygenase 1 (HO-1) and mitochondrial reactive oxygen species (ROS), inhibit glutathione peroxidase 4 (GPX4), lead to ferroptosis, and induce follicular atresia (39). Oocyte basonuclin zinc finger protein 1 (BNC1) defects promote acyl-CoA synthetase long chain family member 4, (ACSL4) expression through the NF2, Moesin-ezrin-radixin like (MERLIN) tumor suppressor-yes-associated protein (NF2-YAP) pathway, increase lipid ROS, and induce ferroptosis (40). Sirtuin 3 (SIRT3) loss in trophoblast cells activates AMPK/mTOR and inhibits GPX4, leading to autophagy dependent ferroptosis (41).

## 3 The apoptosis and follicular atresia

**Figure 2:**
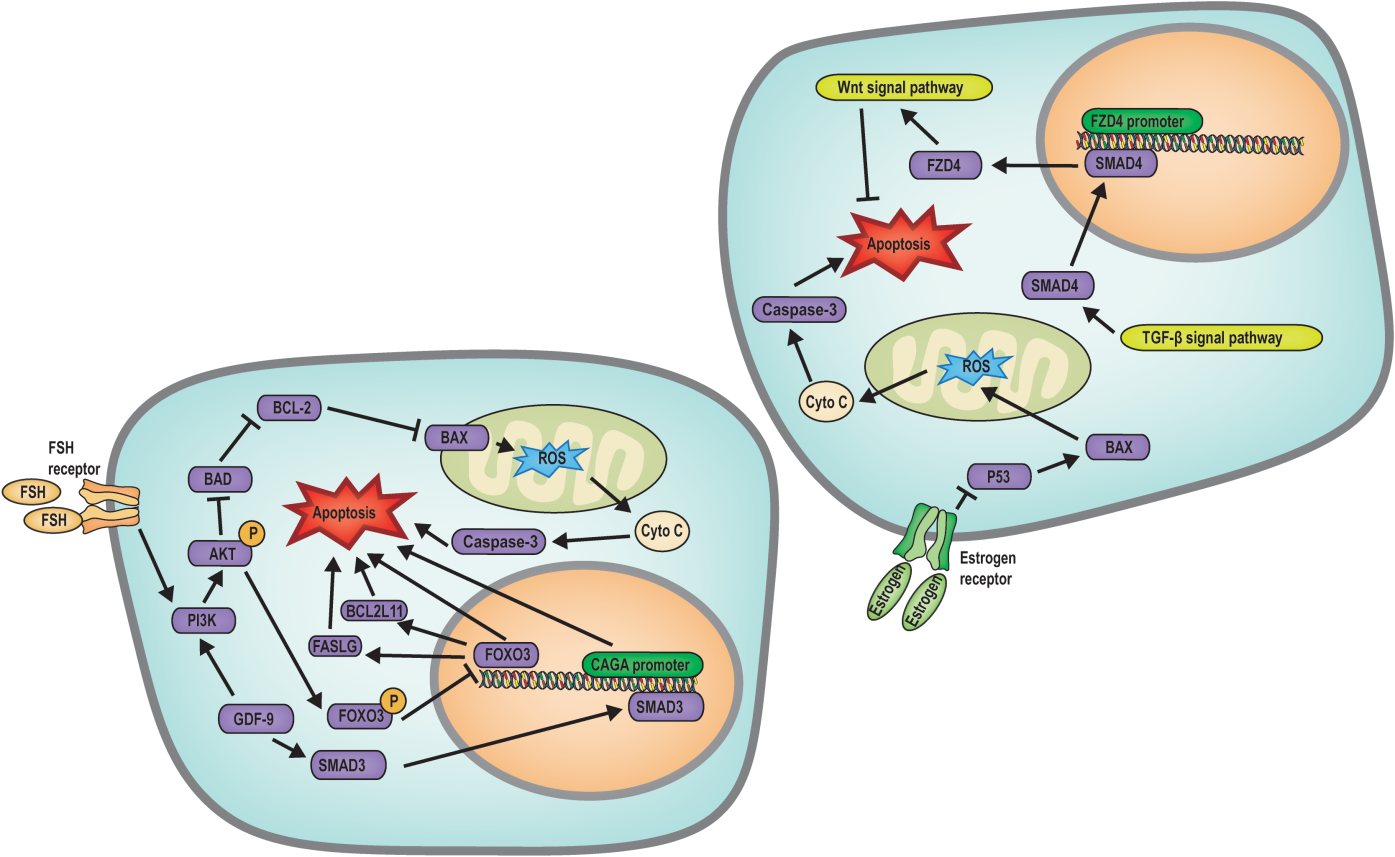
Mechanism diagram of apoptosis regulated follicular atresia. FSH regulates the PI3K/AKT/FOXO3 signaling pathway to inhibit apoptosis, while GDF9 upregulates SMAD3 transcription to promote apoptosis; Estrogen, Wnt and TGF-β signaling pathways inhibits apoptosis.

Apoptosis is a genetically regulated, energy-dependent form of programmed cell death characterized by cell shrinkage, chromatin condensation, membrane blebbing, and the formation of apoptotic bodies. It plays a crucial role in maintaining homeostasis during mammalian embryonic development, particularly in follicular atresia. Many studies have explored the mechanisms underlying granulosa cell apoptosis during follicular development and atresia (**Figure 2**). The following sections provide an overview of these processes.

Biochemical analysis of ovarian tissue has identified nuclear chromatin fragmentation within atretic follicles, a definitive indicator of apoptotic cell death (42). Research has established the expression of BCL-2 gene family members within oocytes, underscoring the pivotal role of oxidative stress in mediating apoptosis (17, 43). Recent investigations have elucidated that a physiological balance of ROS is crucial for modulating follicular development, angiogenesis, and steroidogenesis within the ovary. ROS can directly inflict DNA damage by oxidizing nucleic acid bases and deoxyribonucleoside triphosphates (dNTPs), thereby impairing polymerase activity and diminishing the rate of *in vitro* DNA replication. An imbalance between ROS and antioxidant defenses precipitates oxidative stress (19). In oocytes, phosphorylation of FOXO3 can be promoted through the PI3K/AKT pathway. FOXO3 can regulate the apoptosis of oocytes and granulosa cells through transcription, and phosphorylated FOXO3 is inhibited from entering the nucleus. In this way, the PI3K/AKT pathway regulates follicle atresia and activation. In addition, AKT can regulate the expression of BAX and BCL-2 proteins, inducing mitochondrial oxidative stress and increased ROS levels, leading to the release of cytochrome c by mitochondria, which in turn activates Caspase3 and ultimately leads to oocyte apoptosis (35). This regulatory mechanism can diminish the ovarian reserve function and precipitate follicular atresia.

In addition, GDF9 has been found to inhibit granulosa cell apoptosis by stimulating SMAD3-induced CAGA promoter activity, thereby preventing follicular atresia (44). Furthermore, GDF9 exerts its anti-apoptotic effects in granulosa cells through the PI3K/Akt pathway (36). The transcription factor SMAD family member 4 (SMAD4), a downstream effector of the TGF-β signaling pathway, can directly control frizzled class receptor 4 (FZD4) transcription and promote FZD4-dependent Wnt signaling pathway, thereby triggering granulosa cell apoptosis (45). Hormonal and hormonemimetics factors also play a role in the regulation of this process. FSH activates the PI3K/AKT pathway in granulosa cells and phosphorylates the FOXO3 transcription factor, sequestering FOXO3 in the cytoplasm and preventing it from entering the nucleus. This action inhibits the activation of pro-apoptotic factors such as FASLG and BCL2 like 11 (BCL2L11), thus inhibiting follicular atresia (35). Moreover, estradiol directly suppresses the transcription of pro-apoptotic genes *P53* and *Bax* in granulosa cells, thereby inhibiting apoptosis-induced follicular atresia (46).

Small extracellular vesicles (sEVs) derived from embryonic stem cells have been demonstrated to attenuate the expression of proteins associated with apoptosis. Post-transplantation of sEVs, serum hormone levels normalize, the count of follicles notably increases, and the incidence of apoptotic cells declines. sEVs significantly enhance the proliferation of granulosa cells, upregulate the expression of phosphorylated PI3K and AKT, and stimulate the PI3K/AKT/mTOR pathway, thereby curtailing granulosa cell apoptosis and follicle atresia. sEVs play a role in follicle formation to maturation (47).

Research has indicated that adipose-derived stem cell (ADSC) transplantation can curtail apoptosis in ovarian granulosa cells from follicle formation to follicle maturation and augment the quantity of total, primordial, primary, and mature follicles. ADSCs have been shown to markedly reduce senescence and apoptosis in granulosa cells triggered by the chemotherapeutic agent cyclophosphamide (CTX). At the molecular level, ADSCs curb apoptosis in granulosa cells by modulating the PI3K/AKT/mTOR signaling cascade (48). This may be a method for the treatment of abnormal follicular atresia and the maintenance of follicular activity.

Certain pharmacological agents have demonstrated efficacy in curbing apoptosis-induced follicular atresia and promoting the activation of primordial follicles. Antioxidants, including vitamin C, vitamin E, glutathione, sulforaphane, American cockroach peptide, and resveratrol, have been shown to mitigate cellular oxidative stress, thereby inhibiting follicular atresia. Moreover, anti-inflammatory medications, such as select corticosteroids and nonsteroidal anti-inflammatory drugs, attenuate inflammatory responses and redue oxidative stress levels, consequently inhibiting follicular atresia (49, 50).

The issue of chemotherapy-induced follicular atresia has garnered significant interest in the field of female reproductive health. In addition to the ADSCs, which can mitigate chemotherapy-induced granulosa cell damage via the PI3K/AKT/mTOR pathway, metformin has been demonstrated to prevent cell apoptosis through activate the AMPK pathway, thereby protecting granulosa cells in chemotherapy-induced follicular atresia (51). Furthermore, traditional Chinese herbal medicines such as Angelica sinensis (dong quai), Codonopsis pilosula (dang shen), Astragalus membranaceus, and Cuscuta seed have been reported to modulate BCL-2 family proteins, inhibiting cell apoptosis, promoting follicular development, and reducing follicular atresia (52, 53).

## 4 The autophagy and follicular atresia

Autophagy is a cellular self-degradation process in all eukaryotes, functioning under virous physiological and pathological conditions. This mechanism requires the degradation and recycling of excess or damaged cellular components, including proteins, lipids, nucleic acids, and specific organelles such as mitochondria and peroxisomes. Autophagy is essential for maintaining cellular homeostasis and metabolic balance (54).

**Figure 3:**
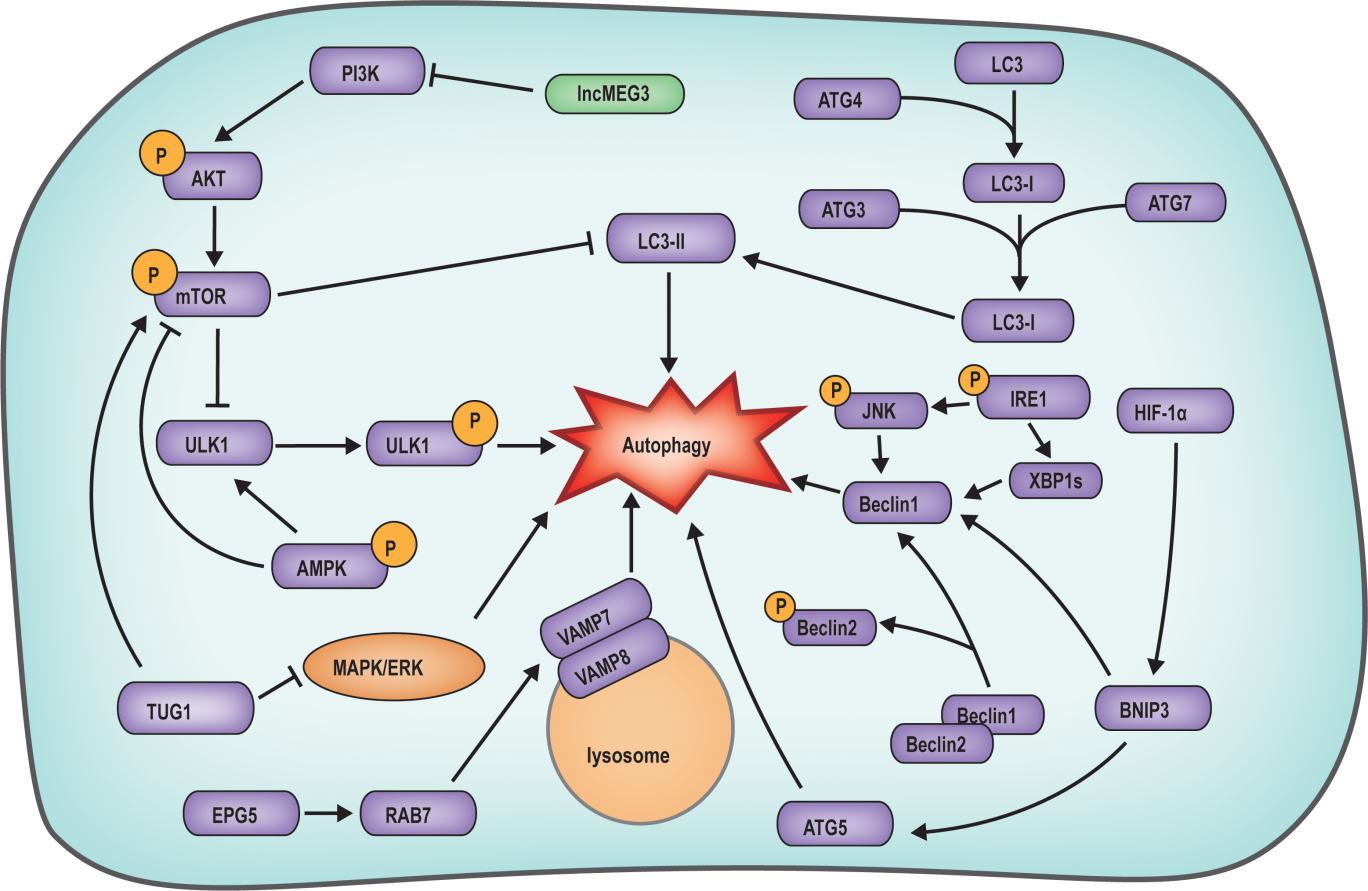
Mechanism diagram of autophagy regulated follicular atresia. The AKT/mTOR signaling suppresses autophagy by downregulating the expression of LC3-II and ULK1. TUG1 in follicular cells suppresses autophagy by inhibiting MAPK/ERK pathway. IRE1 can enhance Beclin1 expression by activating XBP1S expression and HIF-1α phosphorylation, with the latter directly activating autophagy. The expression of EPG5 activates RAB7, which interacts with the VAMP7/VAMP8 complex on the lysosomal membrane, facilitating the initiation of autophagy.

Research has illuminated that apoptosis is not the exclusive determinant of follicular atresia; rather, autophagy also exerts a distinct influence on follicular atresia (**Figure 3**). Autophagy has a dual function, with basal autophagy supporting the survival of granulosa cells by clearing damaged organelles. However, nutrient deprivation or PCOS caused by hormone imbalance can over-activate the AMPK/mTOR pathway, upregulate LC3B and Beclin1, and induce autophagy-mediated follicular atresia (55, 56). Examination of gene expression profiles coupled with bioinformatics analyses has uncovered the upregulation of autophagy-related genes, including autophagy related 4B cysteine peptidase (ATG4B), autophagy related 3 (ATG3), autophagy related 13 (ATG13), and ULK1, during granulosa cell demise, underscoring the potential pivotal role of autophagy in the etiology and molecular underpinnings of follicular atresia. This discovery lays a molecular foundation for the diagnosis and therapeutic intervention of follicular atresia (57).

Investigations have demonstrated that during the primary to antral follicle phase, the autophagy-related protein ATG family is involved in the regulation of follicle atresia, and the classic autophagy pathways of autophagy related 5 (ATG5), Beclin1 and LC3 are all activated, and autophagy occurs in granulosa cells, which directly leads to follicular atresia (58). In endometriosis studies, Beclin1, a crucial autophagy protein, typically interacts with dephosphorylated Beclin2. However, phosphorylation of Beclin2 leads to the dissociation of Beclin1, enabling it to directly trigger granulosa cell autophagy. This, in turn, stimulates low-density lipoprotein-induced progesterone synthesis, culminating in follicle atresia (59). Current research indicates that during the primary to antral follicle phase well-characterized signaling pathways, such as PI3K/AKT/mTOR, mitogen-activated protein kinase/extracellular signal-regulated kinase (MAPK/ERK), AMPK, and inositol-requiring enzyme 1 (IRE1), by inducing granulosa cell autophagy, the primordial follicle formation is altered, resulting in a decrease in oocyte count and the occurrence of follicular atresia (60). The expression of maternally expressed gene 3 (lncMEG3) within granulosa cells can also curtail autophagy by regulating the PI3K/AKT/mTOR pathway, thereby restoring granulosa cell proliferation (56). In a study on polycystic ovary syndrome, taurine up-regulated 1 (TUG1) expression was found to inhibit autophagy by obstructing the MAPK/ERK pathway, while concurrently activating the mTOR pathway to suppress autophagy, leading to aberrant follicle growth (61). In a study on autophagy in mouse ovarian cells, it was observed that IRE1 could induce the phosphorylation of its downstream molecule c-Jun N-terminal kinase (JNK) and the expression of X-box binding protein 1 (XBP1) through phosphorylation, subsequently leading to increased expression of Beclin1 and triggering ovarian cell autophagy (62). Additionally, Ectopic p-granules 5 autophagy tethering factor (EPG5) deficiency is correlated with primary ovarian insufficiency due to ovarian failure. EPG5 in granulosa cells induces autophagy by directly interacting with ras-related protein rab-7a (RBA7) and binding to the vesicle associated membrane protein 7-vesicle associated membrane protein 8 (VAMP7-VAMP8) complex on lysosomes, facilitating its transfer to autophagosomes, culminating in follicle atresia (63).

Numerous hormones, biomolecules, and chemicals can induce autophagy in follicular cells by modulating the PI3K/AKT/mTOR pathway. The AKT protein, previously mentioned, not only engages in the regulation of cell apoptosis but is also recognized as a key regulator of autophagy. In the presence of FSH, AKT suppresses granulosa cell autophagy by modulating the phosphorylation of the PI3K/AKT/mTOR signaling pathway, consequently diminishing the expression of LC3B (64). Moreover, melatonin has been demonstrated to modulate autophagy in ovarian granulosa cells via the PI3K/AKT/mTOR pathway (65). Studies on the endocrine-disrupting chemical Bisphenol A (BPA) have revealed that BPA, exhibiting estrogen-like effects, activates the AMPK/mTOR/ULK1 pathway. Phosphorylated AMPK can directly activate ULK1 or indirectly activate it by inhibiting mTOR, promoting granulosa cell autophagy and subsequently precipitating follicle atresia (66).

Some pharmacological interventions have demonstrated efficacy in mitigating autophagy-induced follicular atresia and promoting the activation of primordial follicles. Clomiphene, an orally administered medication, modulates ovarian cell autophagy by targeting the mTOR pathway and LC3, and is utilized in the clinical management of PCOS (67). Metformin, a widely recognized antidiabetic drug, has also been employed in the treatment of patients exhibiting follicular atresia. Studies indicate that metformin ameliorates PCOS by attenuating oxidative stress-induced autophagy via the PI3K/AKT/mTOR signaling cascade (68, 69). Furthermore, research suggests that cyclosporine A activates autophagy associated with hypoxia-Inducible Factor 1-Alpha/BCL2 interacting protein 3 (HIF1α/BNIP3) pathway. Hyperoside protects against ovarian damage and reduced fertility induced by cyclosporine A by inhibiting HIF-1α/BNIP3-mediated autophagy, thereby preserving the follicular reserve (70).

Overall, these observations imply that the modulation of autophagy could present novel therapeutic avenues for the management of follicular atresia. The manipulation of the autophagy pathway using pharmacological agents or bioactive molecules may constitute a promising treatment strategy. Additional investigative efforts are warranted to dissect the intricate interplay between autophagy and follicular atresia, and to assess the feasibility of harnessing the autophagy pathway as a therapeutic target for follicular atresia.

## 5 The ferroptosis and follicular atresia

**Figure 4:**
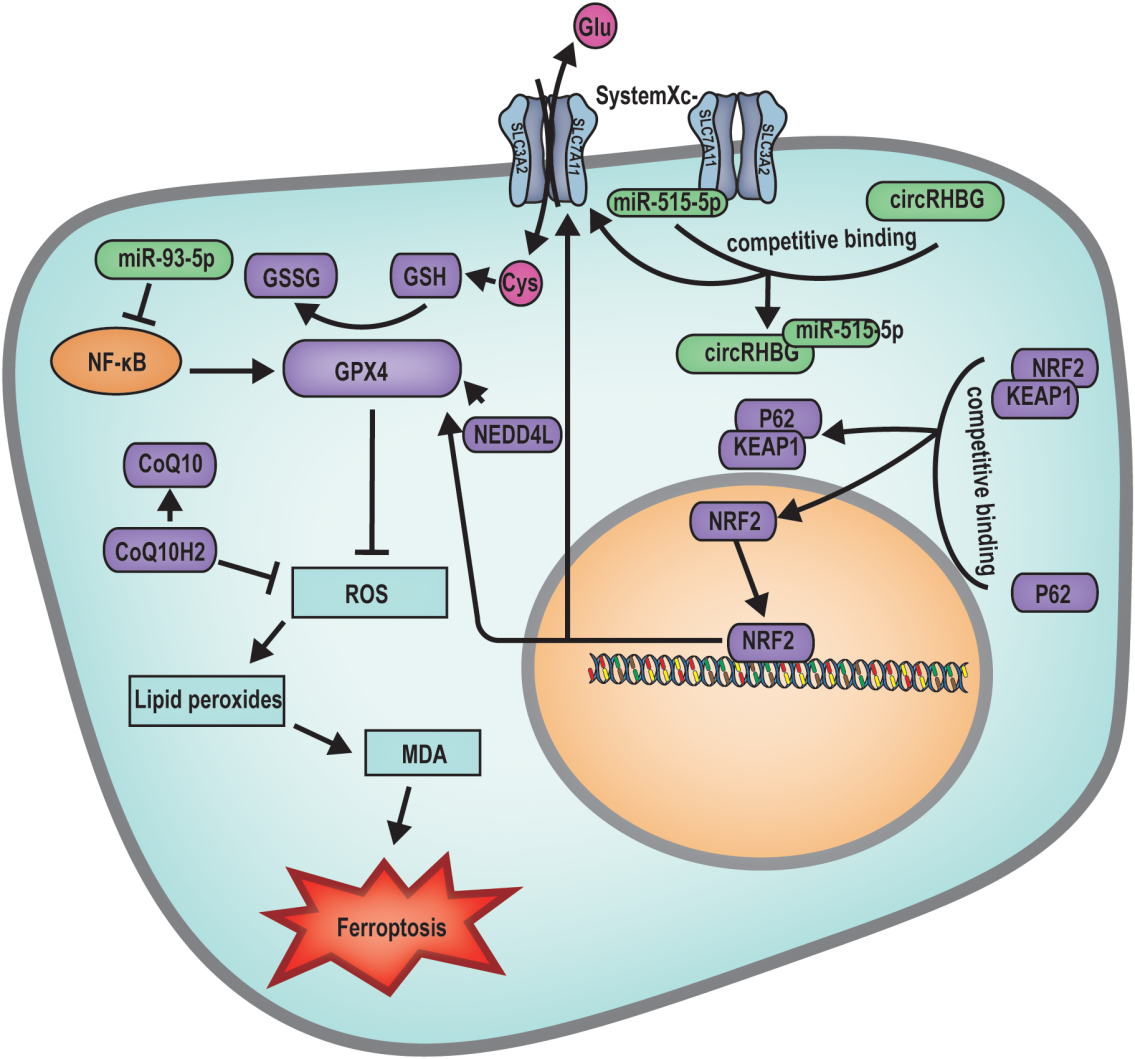
Mechanism diagram of ferroptosis regulated follicular atresia. miR-93-5p inhibits the NF-κB pathway, subsequently suppressing the expression of GPX4, leading to increased ROS production inducing ferroptosis. circRHBG promotes the expression of SLC7A11, enhancing the GSH/GSSG ratio, inhibiting ferroptosis. BNC1 directly participates in the regulation of the NF2-YAP signaling pathway, regulates the transcription of ACSL4. The expression of SIRT3 inhibits the expression of GPX4, facilitating the occurrence of ferroptosis. On the other hand, it can activate the AMPK-mTOR signaling pathway, inducing autophagy-dependent ferroptosis. NEDD4L acts on GPX4 to suppress ROS production, thereby inhibiting cellular ferroptosis. P62 binds to the KEAP1/NRF2 complex, releasing the transcription factor NRF2. Once NRF2 enters the nucleus, it can regulate the expression of SLC7A11 and GPX4, thus inhibiting ferroptosis.

Ferroptosis is an iron-dependent, novel form of programmed cell death that differs from apoptosis, necrosis, and autophagy (39). The main mechanism of ferroptosis involves a decrease in cellular antioxidant capacity and accumulation of ROS due to the direct or indirect impact on glutathione peroxidase by ferrous iron or lipoxygenases. The interaction between ROS and Fe^2+^ causes a Fenton reaction, ultimately resulting in cell death (39, 41). During the developmental progression of female follicles, follicular atresia has been associated with the cellular mechanism of ferroptosis. Current investigative efforts are primarily focused on the diverse molecular triggers of the ferroptosis pathway, which result in excessive ROS production, oxidative stress, and the subsequent destruction of cellular and organelle membranes, culminating in follicular atresia (**Figure 4**).

At the primordial follicle stage, mutations in genes such as *Bnc1* can precipitate ferroptosis, leading to follicular atresia. BNC1 directly modulates the NF2-YAP signaling pathway, and its deficiency downregulates NF2 expression, reduces YAP phosphorylation, promotes YAP nuclear accumulation, triggers increased iron uptake, and enhances lipid ROS production. These changes result in oocyte ferroptosis and ultimately follicular atresia (40). Research has further demonstrated that elevated ovarian SIRT3 expression can induce autophagy-dependent ferroptosis by activating the AMPK-mTOR pathway and inhibiting GPX4 expression, thereby promoting oocyte ferroptosis (40).

During the follicle development stage, research has indicated that a multitude of miRNAs and lncRNAs are implicated in the modulation of ferroptosis-induced follicular atresia. For example, aberrant expression of miR-93-5p within granulosa cells can affect follicle development through multiple mechanisms, including the induction of apoptosis via the nuclear factor kappa-light-chain-enhancer of activated B cells (NF-κB) pathway and the suppression of *Gpx4* gene expression, leading to the accumulation of lipid ROS and subsequent ferroptosis of granulosa cells (71). Furthermore, elevated levels of the circular RNA Homo sapiens RBM39 binding protein 1 (circRHBG) in granulosa cells have been shown to upregulate solute carrier family 7 member 11 (SLC7A11) expression, increase the glutathione (GSH)/oxidized glutathione (GSSG) ratio, enhance GPX4 activity, and inhibit ferroptosis in granulosa cells, thereby preventing follicular atresia (72).

Beyond RNA molecules, various biological factors also contribute to the pathogenesis of follicular atresia. Studies have revealed that dehydro-epiandrosterone (DHEA), ubiquinol CoQ10, and Cleo-20 T3 significantly diminish the expression of genes associated with the intracellular ferroptosis pathway, including transferrin receptor (TFRC), nuclear receptor coactivator 4 (NCOA4), and solute carrier family 3 member 2 (SLC3A2) and elevates GPX4 levels, indicative of ferroptosis suppression and thus the inhibition of follicular atresia (73). Moreover, it has been discovered that NEDD4 like E3 ubiquitin protein ligase (NEDD4L) in granulosa cells can induce ferroptosis by promoting the ubiquitination and degradation of GPX4, culminating in follicular atresia (74).

In the context of ovarian cancer treatment, chemotherapy drugs often inadvertently damage normal cells alongside their intended impact on cancer cells. For example, following exposure to chemotherapy drugs, granulosa cells undergo mitochondrial damage, leading to decreased GPX4 expression, ROS accumulation, and subsequent ferroptosis, which results in follicular atresia (75). Endometrial stem cells (EnSCs) can upregulate NF-E2-related factor 2 (NRF2) expression in granulosa cells, which in turn suppresses ferroptosis in granulosa cells by promoting the SLC7A11-GPX4 axis expression and thereby inhibiting follicular atresia (76).

Certain pharmacological agents and molecular entities have demonstrated the capacity to effectively suppress the ferroptosis pathway, thereby inhibiting the onset of follicular atresia. Iron chelators, such as deferoxamine and hydroxylamine-methanesulfonic acid, can sequester excess free iron ions, thus averting follicular damage (77, 78). The ferroptosis inhibitor, ferrostatin-1 (Fer-1), and endometrial stem cells (EnSCs) have been shown to restore cellular viability by inhibiting ferroptosis, which mitigates the detrimental effects of chemotherapeutic agents on granulosa cells (76). Cyclophosphamide has been reported to induce ferroptosis in ovarian granulosa cells through pathways involving HO-1 and ROS-mediated mitochondrial dysfunction (39).

All, ferroptosis is posited to play a pivotal role in the activation of primordial follicles, the preservation of the primordial follicle pool, and the modulation of follicular atresia (79, 80). Further investigation is imperative to clarify the precise mechanisms through which ferroptosis precipitates follicular atresia and to explore the therapeutic potential of targeting ferroptosis in female reproductive health.

## 6 Outlook and discussion

Ovarian follicle atresia is a common phenomenon in the female reproductive system characterized by impaired or halted follicle development. Physiological follicle atresia is indispensable, its dysregulation underlies major reproductive pathologies (81). This condition has been associated with disorders such as PCOS and POF (82, 83). The current clinical approaches to managing follicular atresia primarily encompass surgical intervention and hormonal therapy. For example, FSH interacts with its receptors to promote the growth and maturation of follicles, thereby potentially ameliorating abnormal follicular atresia (84, 85). Human chorionic gonadotropin (hCG) functions in a manner analogous to luteinizing hormone (LH) by binding to LH receptors, stimulating the ovaries and facilitating the ovulation of mature follicles (86, 87). Estrogens, including estradiol, modulate follicular development and enhance the follicular environment through their action on estrogen receptors (88).

Additionally, adjunctive therapeutics, such as spironolactone, an anti-androgen agent that targets androgen receptors, which can bolster follicular development in patients afflicted with PCOS (89). Insulin sensitizers, exemplified by metformin, indirectly promote follicle maturation by mitigating insulin resistance, thus providing supplementary therapeutic benefits for PCOS management (90, 91). Nonetheless, these treatment modalities have inherent limitations and carry the potential for adverse effects.

Recent investigations have increasingly revealed that cellular mechanisms such as apoptosis, autophagy, and ferroptosis contribute to ovarian follicle atresia (91–95). These processes often coexist; for example, SIRT3 activates both autophagy and ferroptosis in aging ovaries. Consequently, a more profound exploration of the interplay between these cell death pathways and ovarian follicle atresia could elucidate the underlying mechanismsand pave the way for innovative treatment strategies (96, 97). Future research endeavors could focus on the development of targeted therapies or specific pharmaceutical interventions that modulate the molecular underpinnings of these cell death pathways, potentially enhancing fertility rates and improving the quality of life for individuals with ovarian follicle atresia.

In summary, a detailed study of the effects of apoptosis, autophagy, and ferroptosis on follicular atresia can provide a solid foundation for a more comprehensive understanding of the pathophysiological mechanisms underlying this condition and facilitate the discovery of new treatment approaches. This research also offers new perspectives and opportunities for addressing challenges associated with follicular atresia.
